# Notes and News

**Published:** 1894-03-31

**Authors:** 


### Notes and News.

Collected and Communicated.

### MEETINGS OF THE WEEK.

**Royal Asylum of St. Anne's Society's Schools, Redhill: Anniversary Festival Dinner, Salters' Hall, St. Swithin's Lane, Wednesday, April 4th.**

**Rupture Society: Annual General Meeting, Limmer's Hotel, George Street, Hanover Square, Wednesday, April 4th, at 4.45 p.m.**

**Dental Hospital of London, Leicester Square: Special General Meeting, Thursday, April 5th, at 5.30 p.m.**

A hospital for lepers, to which is to be attached a laboratory for the special study of the disease, has been recently established at Rio de Janeiro.

It is stated that red glass is to be tried in some small-pox wards in New York, to test the value of experiments made by two Norwegian doctors—Lindholm and Vinsen, of Bergen—who declare that violet rays in light have much to do with pitting in small-pox, owing to the sensitiveness of the skin to these rays.

The well-known "present with a pound of tea system" is about to be applied to medical journalism in the United States. This idea hails from Ohio. Every subscriber to a new medical journal is to receive a "surprise packet" every three months. The journal is to be further rendered attractive by a series of prize stories of professional bearing.

At a banquet, at Gibraltar, given to Mr. Ernest Hart, Editor of the *British Medical Journal*, it was decided to establish a branch of the British Medical Association at Gibraltar. The next annual meeting of the parent Association is to commence on Thursday, July 31st, and the locality chosen is Bristol. The programme of proceedings is already issued.

Walsall's charities are to be the richer by £20,000 through the will of the late Mr. Henry Boys.

It will appear strange to Englishmen that the Professor of Ophthalmology in Breslau, Dr. Richard Förster, has been made a life member of the Prussian House of Lords. This probably does not mean a practical share in the governing voice of his country, but it places medicine on the highest pedestal of honour—a gracious recognition never yet accorded medicine in England.

The American Medical Association are endeavouring to obtain the assistance of the law to prevent the spread of blindness due to the neglect of the newly-born by nurses and midwives. They suggest that a nurse or midwife should be required to notify to the officer of health any swelling or redness appearing about the eyes of infants within a fortnight of their birth. Failure to notify to be punished by fine or imprisonment.

Leeds is certainly focusing attention on its medical charities with good effect, and bids fair to set an example to other localities where responsibilities in regard to the sick poor are more lightly thrown aside. The Lady Mayoress of Leeds has held a meeting of ladies, resulting in the formation of committees to take charge of the requirements of individual wards, and others to stimulate the interest and augment the contributions of outlying districts.

Some time back we gave a description of the interesting Jenner relics, then on show at Bristol. We are glad to learn that a larger opportunity of seeing them is given to the profession and the public, as Mr. Mockler has brought his collection to London. The collection is now on view at the First Avenue Hotel, Holborn, from 10 to 6. It is free to the medical profession, and to others on payment of one shilling. It is proposed to offer the collection to the Royal College of Surgeons. A subscription list will be opened in connection with the exhibition, the proceeds of which will be devoted to poor members of the Jenner family.

Before the full value of potassium permanganate as an antidote to morphia poisoning can be admitted, it is asserted that a different test to that demonstrated on his own person by Dr. Moor will have to be applied. It is held by many medical men that permanganate must come into direct contact with the morphia to effect the result credited to it. Hence, when immediately administered, it is likely to prove effectual, but where the poison has already entered the blood the antidote is regarded as very doubtful in effect. Since the publication of Dr. Moor's discovery permanganate was given to a man having taken three grains of morphia. The antidote was successful, but we are not told how long had elapsed between the dose and the antidote.

